# 

**Published:** 1853-10

**Authors:** 


### CONTENTS OF VOLUME VII.

Answer to Dr. Drake's Interrogatories....... 1
A Bird's Eye View of Medico-Dental Practice. By Dr. W. A. Pease,....... 19
A few Suggestions to Young Men. By Dr. J. F. Johnston,....... 45
An Extraordinary Case of Hemorrhage,....... 65
American Journal of Dental Science,....... 72
Answer to Dr. Drake's Interrogatories—Concluded,....... 81
A Maxim for the Dentist. By Dr. John Harris.......111
Application of Artificial Teeth, by the Auroplastic Principle. By Edward Truman. 124
A Remedy for the Warping of Plates.......153
A New Work for the Dental Profession.......154
An Inaugural Dissertation on Intra-Stomatic Galvanism. By Dr. G. Watt,.......157
Application of Artificial Teeth by the Auroplastic Principle,.......209
A Dissertation on the Diseases of the Dental Pulp. By Dr Chapin A. Harris,.......227
Application of Gutta-Percha to Pivot Teeth. By Dr. Richardson,.......238
A Few Hints for Young Dentists. By Geo. L. Paine, D. D. S.,.......241

Clasps. By Dr. B. T. Whitney,....... 34
Crystal Gold for Filling Teeth. By Dr. J. Taft.......113
Crystal Gold for Filling Teeth. Editor,.......151
Correspondence.......256

Dentistry in France,....... 62
Dental News Letter,....... 72
Dr. Bigelow. Dr. J. A. Cook,....... 74
Dental Exhibitions. By a Dental Surgeon,....... 95
Dental Abstracts.......136
Drs. Taft & Watt.......156
Dr. Charles Bonsall's Address.......194
Dr. T. W. Baxter's Combined Blow-Pipe and Forge,.......204
Dental Abstracts,.......210, 260

Effects of Chloroform on the Blood,....... 67
Extraction of Teeth. By Dr. J. Taylor.......102
Extract from Dr. J. Allen's Valedictory Address.......172
Editorial.......262

Forceps for Extraction of Teeth.......155

Inflammation of Dentine or Tooth-Bone....... 57
Iowa Medical Journal,....... 73

Letter from Dr. A. M. French....... 53
Letter from Dr. C. P. Culver,....... 56
Letter from Prof. H. R. Smith.......148
Letter from Dr. A. W. French.......149
Letter from K.......150

Mortality of Children from First Dentition....... 61
Method of Making Sets of Teeth with Continuous Gums. By Delabarre,....... 68
Minutes of the Annual Meeting of the Ohio College Dental Association of Dental Surgeons.......184
Mndi Mission,.......213

Method of Directing Second Dentition. By Dr. James Taylor,........................245
Non-Fatal Accidents from Anæsthetic Agents. By W. H. Mussey, M. D.,...........130
New York Society of Dental Surgeons...........................................153
Nitrate of Silver as an application to the Teeth..............................204
Nitrate of Silver. By Dr. Geo. Watt,..........................................235
New York Exhibition of the Industry of all Nations............................120
New Dental Depot..............................................................154
Our Advertising Department.................................................... 74
Ohio Dental College Association...............................................155
Obituary of Dr. Josiah Flagg,.................................................155
On the comparative periods of decay of the Bicuspid and Molar Teeth. By Dr. W. A. Pease,.....199
Ohio College of Dental Surgery................................................230
Practical Thoughts on Teeth Drawing, By Dr. C. T. Cushman,.................... 39
Do. do. do. do. .............................................................216
Preparing the Mouth for full sets of Teeth.................................... 60
Prepared Gold for Filling Teeth............................................... 72
Proceedings of the American Society of Dental Surgeons,....................... 89
Payments for the Register.....................................................155
Professor H. R. Smith's Valedictory...........................................172
Periostitis. By Dr. D. B. Whipple,............................................215
Regulation of the Teeth. By Dr. T. W. Evans,..................................221
Reproduction of the Inferior Maxillary Bone................................... 64
Replacement of Teeth.......................................................... 65
Reply of the Graduating Class to Professors Smith and Allen's Valedictories. By Dr. G. Watt,.....178
Restoration of Hearing by Artificial Teeth,...................................212
Supernumerary Deus Sapientiæ. Dr. Richardson,................................. 50
Swagging Plates,.............................................................. 60
Subscribers,.................................................................. 73
Springing of Plates. By Dr. Crane............................................. 92
Sponge Gold. By W. H. Dwinelle, M. D., D. D. S.,..............................245
Teeth Swallowed,.............................................................. 73
The Present number of the Register,........................................... 74
The American Society of Dental Surgeons.......................................152
The Mississippi Valley Association of Dental Surgeons.........................155

### CONTENTS OF THIS (JULY) NUMBER.

### ORIGINAL COMMUNICATIONS.

Nitrate of Silver. By Geo. Watt, M. D., D. D. S.,............................235
Applications of Gutta-Percha to Pivot Teeth. By J. Richardson, M. D., D. D. S.,...238
A Few Hints for Young Dentists. By Geo. L. Paine, D. D. S.,...................241
Method of Directing Second Dentition. By James Taylor, M. D., D. D. S.,.......245
Practical Thoughts on Tooth-Drawing. By C. T. Cushman, D. D. S.,..............216

### SELECTED ARTICLES.

Regulation of the Teeth. By T. W. Evans, M. M., D. D. S......................221
A Dissertation on the Diseases of the Dental Pulp, and their Treatment. By Chapin A. Harris, M. D., D. D. S.,.....227
Sponge Gold. By W. H. Dwinelle, M. D., D. D. S.,..............................245
Dental Abstracts,.............................................................250
Correspondence,...............................................................256
Editorial,....................................................................262

### SUBSCRIBERS.

We have many subscribers who are far in arrears. We are satisfied this is more from neglect thau any thing else. We shall shortly remit bills to all such, and hope they will attend to them promptly. When such things pass on from year to year, many will forget to what time they have paid. Those in the profession who receive the Register twice consecutively, are considered subscribers, unless they return the last number, and in such manner as we shall know from whom returned.

### TEETH SWALLOWED.

In our last, we noticed the case of a gentlemen having swallowed three teeth attached to a gold plate. These in a few weeks were discharged, having passed through the entire alimentary canal, and showed no evidences of digestion. The gold was of good quality. The teeth were two front and a lateral incisor attached to a bicuspid on each side.

### OUR ADVERTISING DEPARTMENT.

The Register has been increasing in circulation throughout the West and South, until we are now justified in saying, that it would afford to the Eastern as well as Western manufacturers of Dental Instruments, Materials, etc., a very advantageous advertising medium, and one which we are convinced would pay four fold to those in the trade.

We have made arrangements for enlarging this department. Our terms are as follows: Five lines or less, one year, $5.00; over five lines to a fourth of a page, $10.00 per year; half page, $20 00; whole page, $30 00.

Gentlemen advertising by the year, will receive the advantage of a very large gratuitous circulation of the Register once a year, in which their advertisements will appear.

---

### THE PRESENT NUMBER OF THE REGISTER.

Owing to a press of work on the Publisher's hands for the last month, the Register has been unavoidably delayed. This we hope will not occur soon again.

The article on extraction of teeth which has appeared in the two last numbers of the Register, will be resumed in the next. The present number contains an interesting article on the same subject from Dr. Cushman.

Answers to the remaining questions of Dr. Drake will appear in the next No.

### NINTH ANNUAL ANNOUNCEMENT

OF

### THE OHIO COLLEGE OF DENTAL SURGERY.

**Session of 1853–'54.**

The regular course of lectures in this institution, commences the first Monday of November, and closes on the third Wednesday of February.

### FACULTY.

JAMES TAYLOR, M. D., D. D. S.
*Professor of the Principles and Practice of Dental Surgery.*

THOMAS WOOD, M. D.,
*Professor of Anatomy and Physiology.*

J. B. SMITH, M. D.,
*Professor of Pathology and Therapeutics.*

JOHN ALLEN, D. D. S.,
*Professor of Operative Dentistry.*

H. R. SMITH, D. D. S.
*Professor of Mechanical Dentistry.*

G. WATT, M. D.,
*Lecturer on Chemistry,*

### EXAMINING COMMITTEE.

DENTAL. A. S. TALBERT, D. D. S., of Lexington, Ky.
W. H. GODDARD, D. D. S., of Louisville, Ky.
H. E. PEEBLES, D. D. S., of Lexington, Mo.

MEDICAL. PROF. MENDENHALL, M. D., of Cincinnati, O.
ISRAEL DODGE, M. D., of Cincinnati, O.

The Faculty in issuing this their Ninth Annual Announcement, take pleasure in stating to the Profession that the Institution is based upon a firm foundation, being chartered by the state and owned and governed by stockholders (most of whom are regular Dental Practitioners) in connection with a board of Trustees. In the reorganization of the Faculty, the Profession have every assurance that the interest of the school has been consulted and that the course of instruction will be such as to meet the wants of the dental student.

A building suitable in all the conveniences for a Dental College is owned by the Profession and dedicated to the advancement of dental science.

The library is increasing in useful and appropriate books, and a fund is secured for the continued increase of this and such apparatus as may be needed.

The Professor of Mechanical Dentistry has made arrangements to open the laboratory and infirmary, by the middle of October. Every facility will be afforded the student, and every thing requisite furnished, but such materials as are of a perishable nature.

Tickets for the course, $100,00 ; Matriculating Ticket, 5,00 ; Diploma Fee, $25,00. Good board can be had at from $2,50 to $3,00 per week.

JAMES TAYLOR, *Dean.*

### Baltimore College of Dental Surgery.

### Fourteenth Session, 1853-'54.

CHAPIN A. HARRIS, A. M., M. D,
Professor of Principles and Practice of Dental Surgery.

THOMAS E. BOND, Jr., A. M., M. D.,
Professor of Special Pathology and Therapeutics.

WASHINGTON R. HANDY, M. D.
Professor of Anatomy and Physiology.

ALFRED A. BLANDY, M. D.,
Professor of Operative Dentistry.

PHILIP H. AUSTEN, A. M., M. D.,
Professor of Mechanical Dentistry.

REGINALD N. WRIGHT, A. M., M. D.,
Lecturer on Chemistry and Metallurgy.

The size and convenient arrangement of this new building (corner of Lombard and Hanover streets). in connection with their greatly extended means and improved plan of instruction, enable the Faculty of this, the oldest of Dental Colleges, to offer to ihe students of Dentistry unsurpassed facilities for a complete course of theoretical and practical education.

The lectures on Anatomy, Surgery and Physiology will be amply illustrated by models, drawings and preparations, and by operations upon the living and dead subject. The dissecting room will be at all times well supplied. These Lectures, together with those upon Pathology, Therapeutics and Materia Medica, will fully instruct the student in the general principles of each department, dwelling with especial minuteness upon all that relates to the art and science of Dentistry.

The Lectures on Chemistry and Metallurgy will be illustrated by a very valuable apparatus, recently purchased at great expense by the College.

In connection with the Lectures in the Practical Department, is a large Mechanical room, fitted up completely with all the necessary appliances, and an Infirmary, which has an extensive and constantly increasing practice. The work of the Infirmary and Mechanical rooms will be performed by the students under the immediate direction of the professors in charge. A confident self-reliance is thus acquired, which could not be gained by any amount of mere oral or demonstrative teaching.

Demonstrations will also be given in the manufacture of artificial block teeth.

The regular course of Lectures will commence on the 1st of November, and close the 1st of March. The Infirmary, Mechanical and Dissecting rooms will be open on the 1st Monday in October, during which month there will be occasional lectures from each of the Professors.

It is important that every student should, if possible, be present during this preliminary month. The Infirmary will continue open during the summer for the benefit of those students who remain in the city between their first and second course of lectures.

Candidates presenting themselves for examination, must have attended two full courses of lectures. Certificates of four years' dental practice, or of one full course in any respectable Dental or Medical College, will be received as equivalent to the *first* course.

Tickets for the Course, $110. Dissecting Ticket, (optional), $10. Diploma Fee, $30. Matriculation Fee, $5.

Text Books. Harris's Principles and Practice of Dental Surgery, 5th Edition; Bond's Dental Medicine, 2d Edition; Handy's Anatomy; Carpenter's Physiology; Mutter's Liston's Surg ry; Jourdain's Surgical Diseases of the Mouth; Mitchell's or Dunglinson's Therapeutics and Materia Medica; Fowne's, Graham's or Turner's Chemistry; Wood's or Watson's Practice of Medicine.

P. H. AUSTEN, Dean,
84 *Sharp Street, Baltimore.*

### CONTENTS.

### ORIGINAL COMMUNICATIONS.

Answer to Dr. Drake's Interrogatories........1

Mortality of Children from first Dentition,........61

Dentistry in France,........62

Reproduction of the Inferior Maxillary Bone,........64

Replacement of the Teeth,........65

An Extraordinary Case of Hemorrhage,........65

Effects of Chloroform on the Blood,........67

Method of making Sets of Teeth with continuous Gums, as practiced by C. F. Delabarre, 68

### EDITORIAL.

Prepared Gold for Filling Teeth,........72

American Journal of Dental Science,........72

Dental News Letter—Editorial Change,........72

Iowa Medical Journal,........73

Subscribers,........73

Teeth Swallowed,........73

The Present Number of the Register,........74

Thompson's Compound Self Adjusting Blow Pipe,........74

Our Advertising Department,........74

Dr. Bigelow,........74

Dr. Jacob A. Cook,........74

### RECEIPTS FOR THE REGISTER.

J. A Weaver, Batavia, Ohio, vol. 7,.....$2
C. C. Beilharz, Tiffin, vols. 3, 4, 5 & 6... 8
A. S Talbert, Lexington, Ky., vol. 5 & 6.. 4
Y. W. Lewis, Canton, Miss., vol. 4,..... 2
Robbins, McGill & Co., Meadville, Pa., vols. 5, 6 & 7,........ 5
John G. Harnill, Georgetown, Ky., vol. 6, 2
J. A. Kenicett, Chicago, Ill., vols. 4, 5 & 6, 6
J. Knapp, New Orleans, vols. 5 & 6,..... 4
J. P. Norman, Rockford, Ill., vols. 6 & 7, $3
J. P. Dunlevy, Pittsburgh, Pa., vols 5&6, 4
R. M. Kenedy, Logansport, Ia., vol. 6,.. 2
C. F. Minter, Bolivar, Tenn., vols. 6 & 7, 3
R. Coats, Otsego, Mich., vol. 6,........ 1
H. Clagget, Georgetown, Ky., vol. 6,.... 2
N. B. Stiff, Xenia, O., vol. 5,........ 2
W. C. Pennock, Bellefontaine, O., vol. 7, 2

